# The New York Institute of Stomatology

**Published:** 1903-03

**Authors:** 

**Affiliations:** The New York Institute of Stomatology


### Reports of Society Meetings.

### THE NEW YORK INSTITUTE OF STOMATOLOGY.

A REGULAR meeting of the Institute was held on Thursday evening, November 6, at the office of Dr. J. Morgan Howe, No. 12 West Forty-sixth Street, New York, the President, Dr. Howe, in the chair.

The minutes of the last meeting were read and approved.

### COMMUNICATIONS ON THEORY AND PRACTICE.

Dr. Eames, of Boston, presented an apparatus for removing the débris from instruments with one hand during operations. It consisted of a cup, at the bottom of which was placed a rubber mat, the cup to be filled with some antiseptic solution.

Dr. T. Adams Bishop presented a tooth (second bicuspid) which he had recently extracted and which showed a completely calcified pulp in position.

Dr. F. Milton Smith presented a method for quickly repairing a bridge from which a facing has been broken. The pins in the bridge are left in position. A cross-pin plate tooth is selected, the pins ground away, and a dovetail cut in the porcelain with a thin stone. The tooth is set with cement. He said that it made a good temporary repair.

Dr. C. O. Kimball called the attention of the members of the Institute to Dr. S. A. Hopkins's book, published by Appleton as one of their health primers. He said that the book ought to be in the hands not only of every dentist, but of every mother and all who have the training of children. Its especial value was contained in the instructions given in two or three chapters as to how the teeth of children may be influenced by proper attention to food and daily care. Dr. Kimball believes that it is a work of very great value.

A paper entitled "Dental Bridge and Pier Construction," by Dr. William Slocum Davenport, of Paris, was read by Dr. E. A. Bogue.

(For Dr. Davenport's paper, see page 161.)

Dr. E. A. Bogue also mentioned the following details of Dr. W. S. Davenport's methods:

Impressions must be taken and the models studied from the *lingual* side, as well as from the buccal, before one can be sure what sort of apparatus can be made.

The tendency is for teeth that are bridged to spread apart, hence it is important that the articulation of the natural teeth should hold the abutments in place, if it can be done.

This is *the* important thing to consider before deciding what to construct (it is generally the last thing considered, if it be considered at all, hence the frequent failures).

Asbestos powder, wet up with alcohol, is used to set the parts into solder, as much time is saved by this process.

If teeth are to be used in a bridge, grind them into place before backing; grind all occlusal notches, or cut cusps, or imitation cavities, and then line with thin pure gold.

Invest on the plaster and sand model, and when set remove wax with hot water. Solder all of it at once, and the solder will flow to the bottom of the plaster mould (platinum will not flow there), because the teeth having been backed to the edges, the solder will flow from edge to edge and leave a smooth surface requiring little filing or polishing.

Dr. E. H. Babcock asked whether it was advisable to have pieces of bridge-work rest on the gum or not, also whether it was better to devitalize teeth that were to be used as piers.

Dr. Bogue replied that one of the reasons Dr. Davenport had worked out this system was to preserve the pulp alive. One of the specimens passed around was a bridge resting on the gum for a considerable surface, showing that Dr. Davenport regarded this advisable in some cases.

Dr. S. E. Davenport highly commended the essayist for the originality of methods described in the paper and proved by the diagrams and models exhibited. He said that while the Institute was disposed to occupy a very conservative position concerning ordinary bridge-work, it could but receive with favor such an exhibition as had just been made of Dr. W. S. Davenport's methods, in which bridge-work was but an incident, used not only to increase the masticating surface, but to hold the natural teeth in the new positions into which they had been moved, many models showing also that the bite had been opened to the great improvement of occlusion.

Dr. L. C. Leroy said that there was much to be commended in the paper; but there were some undesirable features, such as the molar with the open masticating surface and the ball-and-socket pier which has been discarded. In one case Dr. Davenport had combined regulating with the bridge-work.

Dr. J. B. Locherty remarked that we were greatly indebted to Dr. Davenport for his paper, because the models described and illustrated were those of cases which had been in practical operation for several years and had satisfactorily stood the test of time.

Dr. L. Ashley Faught, of Philadelphia, expressed his interest in the paper and was sure that a closer study of the models would be of value. He would have doubted the future of some of the bridges if the question of occlusion had not received such careful attention. Dr. Faught, after experimenting with the various kinds of modelling compounds, has settled down to that made by Ash & Sons for taking his impressions, and finds that if properly used it will give very accurate results.

Dr. F. Milton Smith said he was delighted with the paper and illustrations. He believed in bridge-work and thought that a bridge carefully and judiciously made was one of the best things we could do for our patients. That bridge-work done with the idea of correcting articulating surfaces and moving into position certain teeth that were out of place was very valuable. Speaking of burnishing the crown around the neck of the tooth, Dr. Smith said that he had not been able to do it to his satisfaction. He has been extremely conservative about devitalizing teeth to be used as piers, but at times has wished that he had been a little more radical. His plan for treating teeth with bell-shaped crowns was to make a cap that did not extend down to the gum. Although this does not make quite so firm a support, it gives him a chance to examine the tooth under the cap.

Dr. E. A. Bogue said that he happened to know that Dr. Davenport had in mind, when he spoke of burnishing the crown around the necks of teeth, the badly exposed teeth of an elderly person where the margin of the crown could be burnished in around the three roots. Dr. Bogue said that he was adverse to bridge-work which did not allow for the normal mobility of the teeth, but he found that in the bridges devised by Dr. William Davenport not only this had been accomplished, but, as Dr. S. E. Davenport has mentioned, the idea of regulating deformed conditions also. Therefore he had urged Dr. William Davenport to bring the subject before the Institute.

Dr. F. Milton Smith said that in taking impressions for crowns with modelling compound it was his habit to soft solder a band of copper a little larger than the tooth and to use this as a cup. In this way he was able to get perfect adaptation.

Dr. Clinton Wright Strang, of Bridgeport, Connecticut, was called upon to speak of his trip to the International Dental Federation, at Stockholm, Sweden. He stated that he had enjoyed his trip very much, and expressed his pleasure in meeting many of the American dentists who are practising abroad.

Dr. E. A. Bogue was then asked to present his report as the Institute's delegate to the meeting at Stockholm.

### REPORT OF DR. BOGUE AS A DELEGATE TO THE MEETING AT STOCKHOLM, SWEDEN.

Mr. President,—Agreeably to the request of your executive committee, I present you this evening an incomplete report of my doings as your delegate to the International Dental Federation, held in Stockholm last August. Lest it should not be perfectly known by all the gentlemen present what the International Federation is, I will go back two years to August, 1900, and recall the International Dental Congress held in Paris on the occasion of the great exposition there. This congress was composed of about twelve hundred members; it followed upon the heels of the International Medical Congress, which contained a dental and oral section, at which something like one hundred and fifty members were present. The organizing qualities of the men who conducted the Dental Congress it is not necessary to enlarge upon, as the twelve hundred members did that sufficiently. It was thought upon the conclusion of this congress that, in view of its success, an effort might at some future time be made to hold another; and in order that this other might be regarded as a lineal descendant, it was voted by the officers and members present at the last session of the congress, held on August 14, 1900, that a body should be organized, to be called the "Fédération Dentaire Internationale," and that this name should be in French, to be abbreviated by the initials F. D. I., for short; that this body should be composed of all the national committees and the officers of the closing congress; that the president of the congress, M. Godon, and the secretary-general,

M. Sauvez, should continue to act as the president and secretary of the federation; that it should be represented by an executive council composed of nine members, who should be nominated by the then present members of the Third International Dental Congress then assembled, and that the powers of this committee should expire at the opening of the Fourth International Dental Congress, which it is charged to organize at such time and place as might to it appear best in the future. This executive council at its first session proceeded to organize an International Commission of Education, of which Dr. Brophy, of Chicago, was made chairman.

The second session of the F. D. I. was held in London, England, beginning on August 4, 1901, and continuing in Cambridge on August 7, 1901, and was marked by the formation of an International Commission of Public Dental Hygiene. Sir Michael Foster acted as temporary president during the Cambridge meeting. The third session was the one to which you sent your two delegates, and it was held in the city of Stockholm, Sweden, beginning August 13, 1902, and continued three days.

An invitation was sent by the National Dental Association of America to the executive council of the F. D. I., requesting that the Fourth International Dental Congress should be held in 1904, and that the place of meeting should be the city of St. Louis, and the time the month of August. A similar invitation was sent to the American Dental Society of Europe, and this invitation was accepted by both bodies.

A resolution was passed by the International Commission of Education recommending, in substance, that the preliminary examination required of the student wishing to commence the study of dental surgery should be the same as that required for the student wishing to commence the study of medicine, in all countries where educational matters are under the control of the general government; and in those countries where educational matters are not under control of the general government, that the examinations required for a dental student should be the equivalent of those required for the medical student of the countries just mentioned, and that such equivalency should be decided upon by the minister of public instruction.

It was a good deal of a triumph and speaks exceedingly well for the temper and earnestness of the delegates assembled in the Commission of Education of the F. D. I. that they should have been able, after nearly three days of discussion, to agree upon a recommendation of this kind, for it shows a greater harmony among the teaching bodies than could at first sight have been expected, and a greater degree of unity in the requirements that will henceforward be expected from all those wishing to begin the study of our specialty.

PERSONAL EXPERIENCES.

One or two of our members having expressed a wish that I would give my own personal experiences, I shall take pleasure in doing so up to certain point.

There were seven of us on the Steamship "Columbia," of whom Dr. Strang and myself were two. On the way over Dr. Strang grew visibly, because, as he said, every dinner was a banquet, or at least it was the equivalent of many a banquet for which he had paid five dollars, and he was not sea-sick. I also was anxious that the steamer should not get the better of me, but was compelled to forego dinner one day at least.

After a pleasant trip of one week, we reached Hamburg on Friday, going up to the dock at the same moment with the steamer "Blucher," having on board Drs. Guilford and Holly Smith. The trip around Hamburg was a most delightful one, as we found the residence portion of the city was situated along the borders of the river, running out for about four miles, so that on each side of this water the beautiful gardens with their villas in each were sloping down nearly to the water's edge, giving just room for a street or road to pass between the houses and the water. The architecture as well as the horticultural part were both very attractive.

Dr. Griswold issued an invitation to us all to be his guests at dinner and the circus, but your other delegate, Dr. Strang, and myself concluded that we would flock alone, and as I wanted him to have a "circus," I took him to five or six different places of amusement in the course of the evening. His face grew visibly long before we were through, and he asked, with a tone of anxiety in his voice, what his share of the total expense might be, as he wished to liquidate this debt if he had money enough, remarking that he must keep enough to take him home, and that his letter of credit was limited. After long and elaborate calculation I found that he owed me about nineteen cents in our money, which he paid up with a sigh of relief, for I think he fancied it must have been about nineteen dollars.

From Hamburg we all went together to Copenhagen, where we stayed one day, being driven around the city to see the sights that it contains, foremost among which may be counted its commercial advantages, its fine location, and the Thorwaldsen Museum of Sculpture, and the other royal and civic museums and galleries. Our lunch was taken at the Tivoli Gardens, where there is an assemblage of restaurants, theatres, concert halls, and places of out-door sports that I have not seen equalled elsewhere. At this city we joined the Swedish Dental Society, the members of which enjoyed on this occasion half-fare rates over all the railroads of Sweden. At seven o'clock we left Copenhagen for Malmö, which we reached after about two hours and a half ocean travelling. Our numbers had increased by this time to such an extent that we entirely filled one sleeping-coach. In the morning our first experience was getting breakfast at a station about an hour and a half from Stockholm. We were told to go and help ourselves at a bountifully spread table, taking what we pleased and sitting down if we had a chance. The fare was good, and when we came to pay up, every one paid the same,—one kroner, equal to about twenty-six and a half cents of our money,—and upon paying we received a ticket that allowed us to go out—admission free. About nine o'clock we reached Stockholm and our head-quarters, those of us who could got rooms at the Grand Hotel, which was headquarters for the meetings; those who could not, went elsewhere, and your two delegates were placed in a "pension," or boarding-house, which was truly Swedish, very pretty, neat, and clean. After we had been there about a week we discovered that the price of board was six kroners a day, and the gentleman who took us there had been receiving ten with a smile that was childlike and bland; but as he was strongly recommended to us from Chicago, we did not know what to say, so we said it. Breakfast was over in this house when we arrived, and we were politely requested to wait until lunch for our coffee; but we, being willing to try our wings in a strange country, flew out and had coffee, bread and butter, boiled eggs, ham, tongue, sliced cucumbers, with sugar and vinegar, and pickled salmon. However we got all these things Dr. Strang did not know. He thinks it was due to my limited knowledge of Swedish. I knew, on the contrary, that it was the regular breakfast, such as was served at the station before we arrived in Stockholm, and three or four words only brought the whole outfit.

Stockholm is wonderfully clean, bright, and cold. Built mainly of light cream-colored stone, almost white, on eight or ten islands connected by bridges, yet in spots so wide apart that large passenger steamers can lie at their wharves right in the city, and freight-boats only a little way from the centre of town. Passage around the city from place to place is easy,—on foot, in carriages, or in tram-cars, as one will. Hills lay at one side, and quite a table-land on top of the hills, so that a large lake of fresh water is always there, overflowing into the streams and estuaries below. The city is so far from the sea that the tide is not felt, so all this water has the appearance of large rivers or lakes that do not leave an unpleasant mark on their banks.

Our head-quarters were at the Grand Hotel, an enormous and very beautiful building. In the centre of this building is a room that serves for large gatherings, political or social, over many of which the king himself presides, I am told. The king, being a stockholder in the hotel, it seems, was privileged to arrange his rooms so that banquets, balls, public gatherings, with committee-rooms, cloak-rooms, and all the accessories for a convenient and easy holding of assemblages for business or pleasure may be had at short notice. I don't remember ever seeing any more convenient place in which to hold meetings of from one hundred to four hundred persons.

We reached Stockholm on Tuesday morning, got ourselves settled, and attended a meeting of the American Dental Society of Europe in the afternoon. A few of us dined with Dr. Jenkins at the Grand Hotel in the evening, after which we attended a ball given by the Swedish Dental Society and followed by a supper in the rooms before mentioned.

On Wednesday we listened to a most interesting paper from our fellow-member, Dr. W. E. Royce, of Tunbridge Wells, President of the American Dental Society of Europe. (An abstract of Dr. Royce's paper follows this report.) At noon the American Dental Society of Europe invited all its members and guests to lunch at the restaurant in the Grand Opera-House, and in the evening the President and Mrs. Royce gave a reception at the Grand Hotel, followed by a dance and supper.

On Thursday the regular sessions of the American Dental Society of Europe were continued, at which a number of interesting communications were read, and again a lunch offered to all the visitors, and in the evening the annual banquet of the Society.

On Friday the reception of the members of the F. D. I. took place, with speeches from Dr. Christensen, President of the Swedish Society, and Dr. Forberg, our fellow-member, at the Medico-Chirurgical Institute. In the afternoon of Friday sessions were taking place of the F. D. I. and of the American Dental Society simultaneously, these meetings being about half a mile apart; your delegates endeavored to attend both of them, with the usual results, so that when half-past seven P.M. arrived we were quite ready to attend the banquet which was given at the Grand Hotel.

On Saturday there were clinics by members of the Swedish Dental Society and the F. D. I., and our fellow-member, Dr. Brophy, performed an interesting operation for closure of a cleft palate, the patient being an infant of about five months. In the afternoon of Saturday we were invited on board one of those pretty little steamers and taken down to Saltsjöbaden for dinner at the Hasselbachen restaurant. This is the most fashionable bath-resort in Sweden and is about nine English miles from the city; the trip by boat was through the most beautiful scenery and took us between two and three hours. Once arrived at Hasselbachen, we were given a genuine Swedish dinner; as this is a little peculiar, I will try to describe it. On the piazza adjoining the dining-room were numerous sideboards with piles of plates, knives and forks upon them, pickle-jars, and three or four bottles with something strong in them; some of it was schnapps; another bottle was Swedish punch, still stronger, if possible; another, I am told, was gin,—this is mere hearsay. Cold sliced tongue, sausages, ham sandwiches, bread of various kinds, pickles of various kinds—pickled salmon, pickled eel, caviar, sardines, sardellen, sliced cucumbers, Kiehler sprats, perhaps other things—were in abundant quantities on this sideboard. Every one was expected to help himself to the plates and knives if he wished, and whatever eatables he chose,—drinkables as well. Then he sauntered about the piazza, conversing with such friends as he found or giving his most earnest attention to his eating, as pleased him best. This exercise continued perhaps for ten or twelve minutes; when the signal was given, all hands put their plates on the sideboard and repaired to the dining-room adjoining, where a regular *table d'hôte* dinner of five or six courses was duly served, with wine or beer at discretion. At a certain stage in these proceedings, fortunately for one of your delegates, he got out, reached the train, and made for home, where he arrived elated beyond the power of expression, for the rain began to pour in torrents just as he reached his own quarters.

The next morning was Sunday, which was duly observed by our going on board one or two of those boats before spoken of, getting what breakfast we pleased, Swedish fashion, on our way to the Castle of Gripsholm, which we reached about eleven A.M. This is one of the interesting old royal castles of Sweden, containing, they say, the largest and finest collection of portraits in Europe. We passed two hours or more exploring this castle, and on our return to the boat found tables set all around the decks, with awnings overhead, and on our way home again dinner was served at these tables, to the accompaniment of a splendid male quartette singing the songs of Sweden from the time we left Gripsholm until we again reached Stockholm. We landed at a quay a little way from the Grand Hotel, and were taken to see a swimming-match, the culmination of which involved the men going to the fourth story and plunging from there into the water; to our great astonishment, after they had successfully risen to the surface and swam away, about fifteen or twenty girls, from twelve to twenty years of age, followed their example and plunged in from the fourth story. Strange to say, they seemed to enjoy their ducking, and the muscle displayed on both sides of the house was something remarkable and beautiful.

On Monday the F. D. I. continued its labors all day, and on this day we went home to dinner; but on Tuesday, after working still further, it was agreed to have a general dinner at the Grand Hotel, in which about seventy-five joined. Our friend Dr. Barrett rather made the speech of the evening and covered himself with glory as with a garment; but Dr. Guilford lost the occasion of his life. He said that he had seen many of his former friends and pupils practising in Europe, and he was glad to see them again under present circumstances; he said when they left America and went to Europe to live their instructors sent them over like Joseph in Egypt,—he did not specify which of them he meant, and we have been unable to find which ever since. He also said that their instructors had some fears when they came over here to be under the power of kings and emperors, but that they seemed to get along somehow. Dr. Guilford failed entirely to add the corollary of this statement,—viz., that after a time they reached the point of having those kings and emperors under their power, temporarily at least.

The next day, Wednesday, the resolutions mentioned in the early part of the evening, recommendatory of the qualifications for a dental student, were passed, and at one o'clock all the members gathered on the invitation of Dr. Brophy to take a farewell lunch with him at the Grand Hotel, and a half-hour after its conclusion Drs. Brophy, Barrett, Smith and his wife, Gurlee, and Gordon White, under the guidance of Dr. Andridge, started for Russia with one or two other voyagers; I know they survived this tour, the travelling in which was mostly done at night, sight-seeing by daylight, for I saw them later on in Berlin. Dr. Strang and myself tarried in beautiful Stockholm until Friday morning, employing our time in visiting the museums more than we had been able to up to then. On Friday morning we were escorted to a steamer, introduced to the captain, and recommended as distinguished guests to his kind attentions, and we started for a two and one-half days' trip down the Götha Canal to Gothenburg. This canal takes us to the Baltic Sea through a most picturesque journey, partly by lake, partly by river, passing near the Castle of Gripsholm, which we saw on the preceding Sunday; and then for an hour or so we are practically out of sight of land, until at Mem we are at the beginning of the Götha Canal and enter the first of the seventy-four locks that have to be passed between there and Gothenburg. At a good portion of this trip we seemed to be sailing through somebody's back-yard, for the canal is not much broader than the boat on which we are riding. At other points it broadens out into a river or a lake, as the case may be, and in one instance, Lake Nämern, we are once more for a few moments out of sight of land. The locks were so numerous that time and again we were able to get off and walk for an hour or two on shore, and catching the boat when she sailed in one of these locks, just in time for dinner. This dinner, by the way, is like the other Swedish dinner heretofore described. The wonder is that we foreigners lived to tell the tale, but the truth is we grew to like it and would very much like to go again.

The weather was cold and clear; a heavy overcoat was very acceptable. We were favored with moonlight nights and pleasant weather until we reached Copenhagen, which was accomplished on Sunday evening. The place to take supper on Sunday, or on any day, in Copenhagen is at an enormous garden where a splendid orchestra plays during supper- or dinner-time, and when it rains, as it did that evening, they go in-doors to a hall capable of holding at least fifteen hundred people, where we might eat our supper in peace to the accompaniment of music and stay there as late as we choose.

From Copenhagen we passed back by train to Helsenburg, and there were ferried across to Denmark, the train being carried over the few minutes of sea voyage. We landed in Helsenburg, where it is said the tomb of Hamlet is to be found; our English cousins, and some others, make the pilgrimage to this tomb in serious good faith, regardless of whether Hamlet was a creature of Shakespeare's imagination or not. That evening we were landed once more in Copenhagen, and Dr. Kjaer, the dentist to the king, and sometimes to the present queen of England, took charge of us, taking us to his villa five or six miles out of town, overlooking the harbor, to dinner and for an evening's entertainment and supper. Later on we were taken back to that wonderful Tivoli before spoken of, where for an hour we heard most ravishing music exquisitely played.

From Copenhagen we went as directly as we could to Berlin, arriving just as the decorations for our reception and that of the king of Italy the next morning were being completed. We were received after some trouble in a little hotel, Unter den Linden, and the king of Italy and emperor of Germany, with a whole lot of gayly dressed troops passed by next morning bright and early. It was a pretty gay city for two days, and it was then and there that we discovered our Russian travellers on their way home. Shortly after noon we called on our friend and fellow-member Dr. Sylvester, dentist to the emperor, associated with whom is Dr. Watson, who also takes care of the rest of the imperial family. We were seized, put into a fine carriage, and driven all over Berlin, it seemed to us, the whole afternoon; taken back to the hotel to wash and dress, and were told that the carriage would be in front of the house for us by the time we were ready for it; so we dressed and were taken to dinner, where we found a rather gay assemblage, ladies and gentlemen, and were joined later on by another friend and formerly a member, Dr. Young, of Leipsic. What time we got home I will never tell, but next morning at eight the same carriage was ready to take us away down through beautiful woods to Dr. Sylvester's yacht, which was ordered to receive us and take us up and down until we reached Potsdam, where the emperor and the king of Italy would be passing the night and from which they came in their steam launch, saluting us as they went by. We reached our quarters in safety, much edified and delighted, and yet, strange to say, my friend Dr. Strang at this point deserted and went off to Paris, passing by the way of Brussels; what for I never asked; from that on he will have to speak for himself.

For myself, after taking these two or three post-graduate courses from those who control the kings, I went to Dresden, where I was treated once more with royal honors by our friend and fellow-member Dr. Jenkins. Dr. Jenkins allowed me to stand by his chair and learn what I could during five days, and all the time that he was not answering my numerous questions he was showing me the beautiful places in and about Dresden, or treating me to fine dinners, or behaving in a way that I despaired of ever being able to reciprocate in any way.

From this point on I leave your imaginations to run riot as they will. *Ex uno disce omnes.*

### ABSTRACT OF DR. W. E. ROYCE'S ADDRESS ON THE PHYSIOLOGICAL POSSIBILITIES AND LIMITATIONS IN EDUCATION.

Dr. Royce, in the first place, defined education and held that instruction consists in training the organs of special sense. Certain portions of the brain receive and retain facts. Facts thus stored constitute knowledge.

Knowledge should not be looked upon as an end, but as a means to an end.

Knowledge has had to do with the afferent nerves. Education especially deals with the efferent nerves and the organs of expression, for to educate is to draw out the mind.

A mind must be formed before it can be drawn out; knowledge is therefore a prerequisite to education. As the result of instruction is knowledge, so the result of education is skill.

Man alone possesses a creative mind, capable of almost infinite culture.

The brain is said to be the organ of the mind, associated with it are the sensory and motor nerves, the organs of special sense, and the organs of expression.

It is the function of the sensory areas of the brain to receive and respond to the sensations derived from the organs of special sense.

The motor area has furnished a most interesting field for experimentation, and so thoroughly is this region now known that the physiologist is able to cause muscular action in any portion of the body by applying the electrode to the corresponding motor area in the opposite hemisphere of the brain.

Professor Huxley has said that "man alone possesses the marvellous endowment of intelligible and rational speech, whereby he has slowly accumulated and organized the experience which is almost wholly lost with the cessation of every individual life in other animals."

Of the hand is said, "in consequence of the mobility of the thumb, it is what is termed opposible; its extremity can, with ease, be brought into contact with the extremities of any of the fingers; a property upon which the possibility of carrying into effect the conceptions of the mind so largely depends."

In 1891 Waldeyer advanced the theory of the neurone.

This theory, briefly stated, is that the nervous system is made up of innumerable independent units. Each unit consists of a nerve-cell and all its processes. This unit is the neurone, and these neurones are in contiguity but not in continuity; that there is interlacing of the processes but no actual nerve connection.

Both those who uphold and those that oppose the theory of the neurone agree that in early life the neurones are entirely independent of one another.

Experiments have also proved that in early youth use hastens development, want of use causes atrophy. This explains largely the question of so-called muscular education.

It is supposed that the lost time observed in the passage of a nervous impulse is due to a block at each synapse (the term synapse is applied to the interlacing of the processes or dendrons or branches of two or more nerve-cells).

The synapses act like valves preventing impulses passing in the reverse direction.

Reaction time is the name given to the time lost in the block at these synapses and it varies with different individuals and in the same individual according to his physiological condition.

In old age the reaction time becomes slow.

Practice and attention tend, up to a certain point, to diminish the time.

It is the difference in reaction time which has given rise among astronomers to the term "personal equation."

Turning to experiments in music, it was found that the practised player could strike from twelve hundred to nineteen hundred notes per minute correctly, or from twenty to nearly thirty-two per second.

What part of this education consists in shortening the refractory period, what in shortening the reaction time, or the establishing lines of least resistance in the nervous system, and what in the acquired ability of concentrating the energy of many nerve-cells upon a single object, it is impossible at present to say. It is probably a combination of all.

Physiologists have, thus far, failed to define clearly between brain impulses and spinal reflex. In man, therefore, every motion which is directly associated to consciousness must be attributed to the brain.

With these facts in mind let us return to the illustration of the pianist.

The student, at first, slowly studies out one note at a time and seeks for the key indicated by that note. After vigorous mental effort the impression caused by the sight of the note results in a musical sound.

Between the motor area of the brain and the finger there are hundreds of ways where the impulse may go astray and hundreds of byways which should be transformed by use into many thoroughfares and synapses, which through want of use are undeveloped, causing unnecessary delay.

Muscular education is largely the establishment of these lines of least resistance in the nervous system. They constitute the physical basis of habit.

After years of practise these nervous routes become so perfectly established and the impulses pass so easily and so rapidly that the musician is no longer able to count the individual impulses and is only conscious of a single effort.

Many years ago experience taught that which science has confirmed,—viz., that in order that these nerve courses may be brought to their greatest perfection practise must begin in early youth and be continued without interruption.

What measure of success would the pianist obtain who deferred his practical work upon the instrument until he had acquired a classical education and a thorough knowledge of the theory of music?

Watch the finished surgeon or dental operator and see how he illustrates these words.

Think of the versatility of impulse required for his most delicate manipulations. Is the result less wonderful than that obtained by the musician?

By what miracle is the operator to attain, when youth is passed, that which it would be useless for the musician to attempt?

Behind each of these motions is the brain prompting and guiding the most minute details.

Added rapidity or delicacy of touch does not indicate a lower origin of impulse but a higher culture.

Dr. José J. Rojo, of Mexico, said that he was very much pleased at having the opportunity of attending this meeting of the Institute and that his reception by the whole dental body in the United States had been most courteous.

It was moved that the hearty thanks of the Institute be extended to Dr. William S. Davenport for the consideration he has shown us in preparing this paper.

Adjourned.

FRED. L. BOGUE, M.D., D.D.S.,
*Editor The New York Institute of Stomatology.*

